# Cholecystectomy before, simultaneously, or after ERCP in patients with acute cholecystitis: A protocol for systematic review and/or meta analysis

**DOI:** 10.1097/MD.0000000000030772

**Published:** 2022-09-30

**Authors:** Kleyton Santos de Medeiros, Ana Clara Aragão Fernandes, Giuliana Fulco Gonçalves, Camila Vilar Oliveira Villarim, Laura Cristina Costa e Silva, Victor Matheus Câmara de Sousa, Amália Cinthia Meneses Rêgo, Irami Araújo-Filho

**Affiliations:** a Postgraduate Program in Health Sciences, Federal University of Rio Grande do Norte, Natal, RN, Brazil; b Instituto de Ensino, Pesquisa e Inovação. Liga Contra o Câncer, Natal, RN, Brazil; c Department of Surgical, Federal University of Rio Grande do Norte, Natal, RN, Brazil.

**Keywords:** cholecystectomy, endoscopic retrograde cholangiopancreatography, laparoscopic cholecystectomy

## Abstract

Cholecystectomy is the intervention of choice for treating acute cholecystitis; when conservative management does not work, it operates on the patient outside the critical condition. It can be performed together with or after endoscopic papillotomy through endoscopic retrograde cholangiopancreatography (ERCP) when it is concurrent with a situation of cholechodocolithiasis or when there is compression and consequent increase in pressure in the bile duct caused by a calculus jammed in the vesicular infundibulum (Mirizzi’s syndrome), with or without jaundice, fever, and pain in the right hypochondrium (Charcot’s Triad), which can progress to sepsis of biliary origin. This review aims to assess whether the timing of cholecystectomy (before or after ERCP) interferes with the postoperative period and clinical outcome in patients with acute cholecystitis.

**Methods and Analysis:**

By searching the MEDLINE/PubMed, Embase, Web of Science, ScienceDirect, ClinicalTrials.gov, CINAHAL, Latin American and Caribbean Literature in Health Sciences, Scopus and Cochrane Central databases, Controlled Trials Registry Randomized clinical trials will be searched to analyze whether ERCP performed before or after open or laparoscopic cholecystectomy (LC) in patients with acute cholecystitis is beneficial or not, through the analysis of postoperative complications. No language or publication period restrictions will be imposed. The primary outcome will be postoperative complications (postoperative morbidity and mortality). Four independent reviewers will select the studies and extract data from the original publications, with a fifth reviewer in case of disagreement regarding the inclusion or not of particular research in the present review. The risk of bias will be assessed using The Risk of Bias 2 (RoB 2.0) tool, and the certainty of evidence will be evaluated using the grading of recommendations assessment, development, and evaluation. Data synthesis will be performed using the Review Manager software (RevMan V.5.2.3). To assess heterogeneity, we will calculate the *I*^2^ statistics. Additionally, a quantitative synthesis will be performed if the included studies are sufficiently homogeneous.

**Ethics and Disclosure:**

Since the present study will review secondary data, previously published and scientifically validated, it will not be necessary to obtain ethical approval. The results of this systematic review will be published in a peer-reviewed journal.

**Prospero registration number:**

International Prospective Registry of Systematic Reviews (PROSPERO) CRD42021290726.

## 1. Introduction

Acute cholecystitis presents a clinical picture of cramping pain in the right hypochondrium, which may or may not radiate to the dorsum on the same side and epigastrium, fever, present Muphy’s sign, associated with laboratory tests showing leukocytosis with a predominance of polymorphonuclear cells.^[1,2]^

Asymptomatic cholelithiasis or not is part of its genesis, through the presence of gallstones in about 90% of cases. Acute cholecystitis may result from gallbladder disease, which is less prevalent and cannot be overlooked.^[3,4]^

Acute cholecystitis is the most common complication associated with gallstones, more prevalent in women than in men of other age groups, at a ratio of 3:1, known in the surgical scenario as the 4F disease. It affects women over 40 years of age, overweight or obese, of childbearing age, and with established offspring.^[5]^

Currently, conservative treatment is initially chosen, through hospitalization, anti-inflammatory drugs, venous hydration, and antibiotic therapy, since it is a polymicrobial pathology. Such conduct may last until the patient is operated on during the same hospitalization or a posteriori, around 30 days after the resolution of the acute condition.^[6–8]^

However, there is the possibility of complications such as persistence of pain, infection, disease progression, and serious outcomes such as necrosis, gangrene, and gallbladder perforation. In these cases, there is no alternative other than surgical treatment, which should not be postponed.^[9,10]^

When stones are identified in the central bile duct and those stuck in the vesicular infundibulum, cholecystectomy requires complementation with exploring the bile ducts.^[11]^

Endoscopic retrograde cholangiopancreatography (ERCP) is highly sensitive and specific for choledocholithiasis, but its most important use occurs after a confirmed diagnosis of choledocholithiasis, having both diagnostic and therapeutic functions. ERCP can be performed before, during, or after cholecystectomy.^[12–15]^

Patients with choledocholithiasis are diagnosed before surgical treatment or are at high risk of complications, such as those with cholangitis or dilated biliary tree, should undergo ERCP preoperatively.^[13]^

Lower-risk patients may undergo laparoscopic cholecystectomy (LC) with cholangiography and laparoscopic bile duct exploration, depending on the surgeon’s skill and the material available in your hospital.^[16–18]^

Generally, a choledocholithiasis identified but not removed during cholecystectomy requires further ERCP for stone removal. Cholecystectomy must be performed safely, but inflammation resulting from the disease and ERCP manipulation can make the surgical procedure difficult, with increased operative time, greater risk of bleeding, and higher conversion rates than when cholecystectomy is performed electively without prior ERCP.^[19,20]^

Studies show that video LC and ERCP, performed with an interval greater than 72h, present inflammatory changes in the bile ducts, making it difficult to approach the gallbladder and bile ducts by video laparoscopy.^[14–16]^

The standardized surgical treatment for acute cholecystitis is LC, which is associated with better recovery, lower morbidity and mortality, and shorter hospital stay.^[11,17]^

In this scenario, ERCP is configured as a diagnostic and therapeutic means for gallstones. However, it is an invasive procedure that requires technical knowledge and an excellent learning curve to obtain the desired results. There is a risk of complications such as bleeding, pancreatitis, and posterior duodenal perforation (rear window syndrome).^[21]^

Based on the above, there is an extensive discussion about the ideal time to perform ERCP, whether before, during, or after cholecystectomy. In addition, some studies demonstrate controversial evidence that the procedure’s period will cause fewer complications.^[5,7–9]^

Prior ERCP decompresses the bile duct, facilitates clearance of the biliary tree, assists in antibiotic treatment as it drains infected bile, relieves acute symptoms, and reduces the risk of LC being converted into conventional surgery or open surgery. In addition to reducing the operative time, taking with it the risks of complications mentioned above.^[13,18–20]^

It is essential to highlight that the simultaneous performance of cholecystectomy associated with ERCP in the same surgical procedure, in addition to increasing the operative time, can raise doubts for the surgical team in case there are complications inherent to one method or another, a fact that, in addition to increasing the morbidity and mortality of patients is a reason for anguish and doubts for surgeons in the face of this diagnostic and therapeutic challenge, since the procedures were performed concomitantly.^[2–4,16]^

Considering the lack of consensus on the subject, especially when dealing with an acute pathology, which requires effective management on time, the objective of the present review is to assess whether the application of the endoscopic procedure before or after cholecystectomy reduces complications. And the morbidity and mortality of patients with acute cholecystitis.

### 1.1. Review question

Does the early, simultaneous, and late performance of ERCP in patients with acute cholecystitis undergoing cholecystectomy improve morbidity rates?

### 1.2. Objectives

This review aims to assess whether the cholecystectomy timing (before, simultaneous, or after ERCP) would interfere with the postoperative period and its clinical outcome.

## 2. Materials and Methods

The proposed systematic review and meta-analysis will conform to the Preferred Reporting Items for Systematic Reviews and Meta-Analyses guidelines.^[22]^ This protocol is registered with the International Prospective Register of Systematic Reviews (PROSPERO), registration number (CRD42021290726).

### 2.1. Inclusion criteria

This systematic review will include the following studies: randomized controlled trial type studies that showed acute cholecystitis, evaluating CRPE in conjunction with cholecystectomy studies, and experiments involving human adults (age 18).

There will be no language or publication period restrictions. Articles published but not peer-reviewed will not be included in the review.

### 2.2. The PECOT strategy

- Population/Participants: adult patients (age > 18) with acute cholecystitis with associated choledocholithiasis, diagnosed through endoscopic or abdominal ultrasound, intraoperative cholangiography (IOC), or magnetic resonance cholangiography.
- Exposure: Cholecystectomy (Open surgery or Laparoscopic surgery);
- Comparator/control: Patients with cholelithiasis or associated choledocholithiasis, but not with acute cholecystitis, who underwent ERCP at different time intervals; ERCP before, simultaneously, and after cholecystectomy;
- Outcome: Postoperative complications in patients with acute cholecystitis who underwent ERCP at different time intervals; before, simultaneously, and after cholecystectomy (postoperative morbidity rate);
- Types of studies: randomizedcontrolled trial.

### 2.3. Types of patients

Participants of the studies will be adults over 18 years diagnosed with acute cholecystitis and undergoing ERCP at different time intervals; before, simultaneously, and after cholecystectomy. There will be no other age or gender restrictions.

### 2.4. Types of interventions

Studies that described adults diagnosed with acute cholecystitis and treated with Cholecystectomy (Open surgery or Laparoscopic surgery) to compare different time frames with ERCP.

### 2.5. Types of outcome measures

The primary outcome to be evaluated will be the postoperative morbidity rate. The secondary outcomes to be evaluated will be postoperative complications: infection, presence of biliary fistula, length of hospital stay, pain and mortality.^[7–9]^

### 2.6. Patient and public involvement

This work is a systematic review protocol. The research will be performed with a wide and comprehensive search of literature from databases, and individual patient data will not be included. Thus, the authors will not involve patients when setting the search questions and determining the outcome measurements during the design and implementation of the study, and in the dissemination of the results.

The authors made all data underlying the findings described in their manuscript available without restrictions at the time of publication. All data will be contained in the manuscript, and supporting information, that is, summary statistics, means, medians, and variance measures, will be available. One of the priorities adopted will be the non-restriction of public sharing of data.

### 2.7. Search strategy

MEDLINE/PubMed, ClinicalTrials.gov, Web of Science,ScienceDirect, Embase, CINAHAL, Latin American and Caribbean Health Sciences Literature, Scopus and Cochrane Central Controlled Trials Registry. Grey literature will be searched in www.opengrey.eu and Google Scholar. Eligible studies may also be selected from the reference lists of retrieved articles

The medical subject heading (MESH) terms will be: (Choledocholithiasis OR “Common Bile Duct” OR “biliary obstruction” OR Gallstones OR Cholelithiasis OR “Cholecystitis, acute” OR Cholecystitis) AND (Cholangiopancreatography OR “Endoscopic Retrograde” OR “ERCP” OR Endoscopy OR Cholangiography OR “Sphincterotomy, Endoscopic” OR “Endoscopic Papillotomy” OR “Endoscopic Papillotomies” OR “Biliary Tract Surgical Procedures”) AND (Cholecystectomy OR “Cholecystectomy, Laparoscopic” OR “Laparoscopic Cholecystectomy”) AND (“Postoperative Complications” OR “Pain, Postoperative” OR “Post-surgical Pain” OR “Postcholecystectomy Syndrome” OR Hospitalization OR Infections OR “Stay lenght” OR Fever OR “Incisional Hernia”) AND (randomized controlled trials OR Controlled Clinical Trial) (Table 1).

**Table 1:** Medline search strategy.

Search items	
**1**	Choledocholithiasis
**2**	Common bile duct
**3**	Biliary obstruction
**4**	Gallstones
**5**	Cholelithiasis
**6**	Cholecystitis, acute
**7**	Cholecystitis
**8**	Or/1-7
**9**	Cholangiopancreatography
**10**	Endoscopic Retrograde
**11**	ERCP
**12**	Endoscopy
**13**	Cholangiography
**14**	Sphincterotomy, Endoscopic
**15**	Endoscopic papillotomy
**16**	Biliary tract surgical procedures
**17**	Or/ 9-16
**18**	Cholecystectomy
**19**	Cholecystectomy, Laparoscopic
**20**	Laparoscopic Cholecystectomy
**21**	Postoperative Complications
**22**	Pain, Postoperative
**23**	Postcholecystectomy Syndrome
**24**	Hospitalization
**25**	Length of Stay
**26**	Infections
**27**	Fever
**28**	Incisional Hernia
**29**	Or/18-28
**30**	randomized controlled trials
**31**	Controlled Clinical Trial
**33**	Or/30-31
**34**	8 AND 17 AND 29 AND 33

### 2.8. Other sources

Eligible studies can also be selected from the reference lists of retrieved articles. That is, the scope of the computerized literature search may be enlarged based on the reference lists of retrieved articles.

### 2.9. Data collection and analysis

#### 2.9.1. Selection of studies

**Figure 1.**
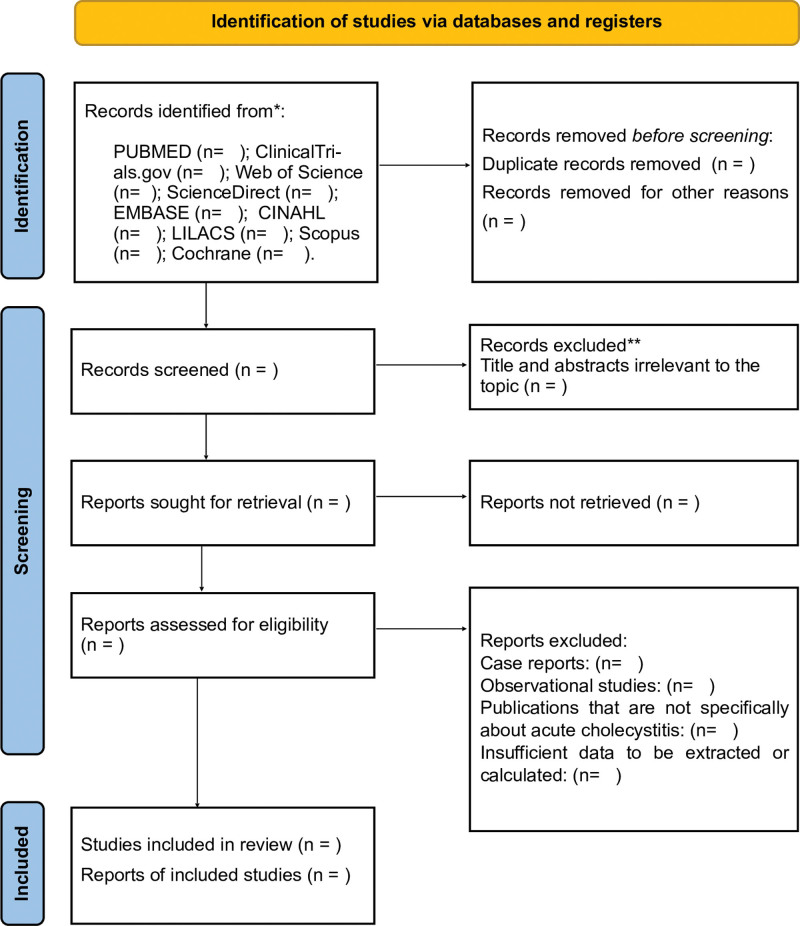
PRISMA flow diagram for systematic review and meta-analysis. PRISMA = preferred reporting items for systematic reviews and meta-analyses.

Four researchers (GFG, CVO, ACF, and LCS) participated in the selection of the studies of interest using Rayyan Software. Titles and abstracts will be read independently and duplicate studies will be excluded. The same authors analyzed the selected texts in order to assess compliance with the inclusion criteria. Um quinto revisor, KSM, will solve the discrepancies. The selection of studies will be summarized in a Preferred Reporting Items for Systematic Reviews and Meta-Analyses flow diagram (Fig. 1).

#### 2.9.2. Data extraction and management

A standardized data extraction form will be developed and tested. Data from each included study will be extracted independently by 2 reviewers (VMC and GFG), and any subsequent discrepancies will be resolved through discussion with a third reviewer (IAF).

The data extracted will include information on authors, year of publication, study location, type of study, main objectives, population, type of surgery, follow-up of participants, surgical intervention, time of ERCP, rates of post-operative, biliary fistula, bile duct injury, hospital length of stay, reoperation rate and mortality. Furthermore, participant characteristics will be extracted (e.g., mean age, gender).

#### 2.9.3. Addressing missing data

In the case of missing data, the authors of this article will contact the corresponding authors or coauthors by phone or email. If we do not receive the necessary information, the data will be excluded from our analysis and will be covered in the discussion section.

#### 2.9.4. Risk of bias assessment

Three authors, KSM, ACAS and APFC, will independently assess the risk-of-bias in the eligible studies using the Cochrane risk-of-bias tool (ROBINS-I).^[23,24]^ Bias is assessed as a judgement (high, low or unclear) for individual elements from 5 domains (selection, performance, attrition, reporting and other).

### 2.10. Assessment of heterogeneity

The heterogeneity between trial results will be evaluated using a standard I2 test with a significance level of *P* < .1. As claimed by the Cochrane Handbook criteria,^[10]^ to assess heterogeneity, we plan to compute the I2 statistic, that is a quantitative measurement of inconsistency across studies. A value of 0% shows that no heterogeneity was observed, whereas *I*^2^ values of ≥ 50% shows heterogeneity; although, the assessment of heterogeneity will only happen if it is appropriate to undertake a meta-analysis.

If the *I*^2^ value is less than 50%, heterogeneity is low, and a fixed-effect model will be used in the analysis. Other, the heterogeneity will be considered high if the *I*^2^ value is 50% or more, and a random effects model will be used. Forest plots will be build to show the study-specific relative risk/odds ratio (OR) estimates and pooled relative risk/OR estimates. We will use forest plots, Eggers’s test and Durval and Tweedie’s trim-and-fill method.

### 2.11. Analysis

Data will be entered in the Review Manager software (RevMan5.2.3). This software allows the user to enter protocols, to complete reviews, include text, characteristics of the studies, comparison tables and study data, as well as to perform meta-analyses of the data. For dichotomous outcomes, we will extract or calculate the OR and 95% confidence interval for each study. Where there is heterogeneity (*I*^2^ ≥ 50%), a random-effect model will be used to combine the studies to calculate the OR and 95% confidence interval.

We will conduct a meta-analysis if we find a pool of included articles with similar characteristics based on the information in the data extraction table. So, if a study is eligible for inclusion in the systematic review but does not provide adequate data for inclusion in the meta-analysis, other study characteristics and results will be summarized narratively to synthesize and tabulate the results.

If the meta-analysis cannot be performed for all or some of the included studies, then sensitivity analyses will be important to explore the robustness of the findings regarding the study quality and sample size, and this is only possible to consider if a meta-analysis is undertaken. This will be shown in a summary table.

### 2.12. Grading quality of evidence

Furthermore, for grading the strength of evidence from the included data, we will use the Grading of Recommendation Assessment, Development, and Evaluation approach.^[24]^ The summary of the assessment will be incorporated into broader measurements to ensure the judgement on the risk-of-bias, consistency, directness and precision. The Grading of Recommendations Assessment, Development, and Evaluation^[25]^ classifies the quality of studies as low, moderate and high. The quality assessment of each study will be independently carried out by 2 authors, and any disagreements will be resolved through discussion (with a third author when necessary).

## 3. Discussion

Currently, treating cholelithiasis associated with choledocholithiasis involves ERCP, followed by cholecystectomy 6 to 8 weeks later.^[17,18]^

LC reduces morbidity, cost, and hospital stay and provides better cosmetic results. However, choledocholithiasis requires retrograde cholangiopancreatography (ERCP), IOC, magnetic resonance cholangiopancreatography, or endoscopic ultrasound for its diagnosis and subsequent treatment.^[19,20]^

In clinical practice, 27% ± 54% of patients with suspected common bile duct stones have choledocholithiasis during ERCP. In addition, 2% ± 15% of patients undergoing endoscopic sphincterotomy have residual stones.^[21]^

The optimal timing for the combined endoscopic/laparoscopic management of choledocholithiasis is controversial in the literature. Some advocate preoperative ERCP followed by LC or LC followed by postoperative ERCP, up to laparoscopic treatment and ERCP in the same surgical procedure.^[7–9,19]^

The diagnostic efficacy of preoperative ERCP for detecting choledocholithiasis is questionable, despite the different screening tests used, and the associated morbidity is not negligible.^[20]^

Using IOC to identify patients who need intraoperative ERCP is an excellent therapeutic strategy, as it reduces the number of unnecessary ERCPs and treats choledocholithiasis simultaneously as cholelithiasis.^[18]^

In the case of acute cholecystitis, the dilemma remains, and few studies address the role of ERCP in patients affected by this surgical emergency. Just as LC is performed in symptomatic cholelithiasis cases, acute cholecystitis cases are treated laparoscopically.^[15,19–21]^

Faced with acute cholecystitis associated with the failure of initial conservative clinical approaches, laparoscopic surgery with or without ERCP is the treatment of choice in many referral centers worldwide.^[18]^

This has led many researchers to investigate the safety and feasibility of ERCP and LC in the same session or during the same hospitalization. Early surgical approaches are becoming more common, even in acute cholecystitis.^[20]^

Recurrent episodes of acute cholecystitis require early cholecystectomy, and common bile duct stones affect about 8% to 20% of these patients. In this sense, LC and ERCP can prevent complications and reduce morbidity by eliminating the source, that is, the inflamed gallbladder (in the case of acute cholecystitis) or bile duct calculus.^[16–19]^

Recent studies have demonstrated the feasibility of a single-step procedure. Rapid technological advances and the accumulation of surgical and endoscopic experience support the use of this approach. The ideal time for treating acute cholecystitis with or without choledocholithiasis is a matter of scientific debate in the era of laparoscopic surgery.^[13,14,21]^

This therapy shortens the interval between ERCP and LC and reduces the recurrence of choledocholithiasis and acute cholecystitis from the time of ERCP to the operation. In this sense, ERCP and cholecystectomy during the same hospitalization are viable and may be the recommended strategy.^[8–10]^

Although there are already standards for the diagnosis and treatment of patients with acute cholangitis associated with choledocholithiasis, the ideal time for cholecystectomy has not yet been defined, especially in the case of acute cholecystitis associated with ERCP, that is, whether it should be performed before, simultaneously with or after LC.^[17–20]^

The 2-stage procedure was performed in patients with choledocholithiasis to reduce inflammation and facilitate cholecystectomy. This approach was adopted before LC became the gold standard, which outperformed open cholecystectomy due to reduced postoperative pain and trauma.^[4–6,8]^

Studies concluded that endoscopic removal of cholechodocian stones and CL performed during the same session were feasible and safe as there were no significant differences in overall morbidity. According to the researchers, single-stage procedures were associated with greater patient comfort and a shorter duration of anesthesia.^[11,19–21]^

When it comes to acute cholecystitis and performing ERCP, randomized controlled studies are scarce, as well as systematic reviews and meta-analyses that address not only the ideal time to complete the procedure, whether before, during, or after LC, as well as complication rates, hospital costs, pain scores, patient satisfaction, quality of life, morbidity and mortality and problem resolution.

## Acknowledgments

The authors acknowledge the assistance provided by the Graduate Program in Health Sciences of the Federal University of Rio Grande do Norte (UFRN) in the undertaking of literary research, and teaching, research and innovation institute of Liga Contra o Câncer, Natal-RN, Brazil.

## Author contributions

ACAF, GFG, CVOV, LCC, and VMCS contributed to the design of this review. ACAF and GFG drafted the protocol manuscript, CVOV, LCCS, and VMCS revised it. ACAF, GFG, and CVOV developed the search strategies, and LCC, and VMCS will implement them. VMCS, LCCS, ACAF, GFG, and CVOV will track potential studies, extract data, and assess quality. In case of disagreement between the data extractors, IAF and ACMR will advise on the methodology and will work as the referee. KSM will complete the data synthesis. All authors will approve the final version for publication.

**Conceptualization:** Kleyton Santos de Medeiros, Ana Clara Aragão Fernandes, Giuliana Fulco Gonçalves, Camila Vilar Oliveira Villarim, Victor Matheus Câmara de Sousa, Amália Cinthia Meneses Rêgo, Irami Araújo-Filho.

**Methodology:** Kleyton Santos de Medeiros, Irami Araújo-Filho.

**Project administration:** Kleyton Santos de Medeiros, Irami Araújo-Filho.

**Supervision:** Kleyton Santos de Medeiros, Irami Araújo-Filho.

**Validation:** Kleyton Santos de Medeiros, Amália Cinthia Meneses Rêgo, Irami Araújo-Filho.

**Visualization:** Kleyton Santos de Medeiros, Amália Cinthia Meneses Rêgo, Irami Araújo-Filho.

**Writing – original draft:** Kleyton Santos de Medeiros, Ana Clara Aragão Fernandes, Giuliana Fulco Gonçalves, Camila Vilar Oliveira Villarim, Laura Cristina Costa e Silva, Victor Matheus Câmara de Sousa, Amália Cinthia Meneses Rêgo, Irami Araújo-Filho.

**Writing – review & editing:** Kleyton Santos de Medeiros, Amália Cinthia Meneses Rêgo, Irami Araújo-Filho.
